# Over-expression of ANP32E is associated with poor prognosis of pancreatic cancer and promotes cell proliferation and migration through regulating β-catenin

**DOI:** 10.1186/s12885-020-07556-z

**Published:** 2020-11-04

**Authors:** Jianwei Zhang, Zhongmin Lan, Guotong Qiu, Hu Ren, Yajie Zhao, Zongting Gu, Zongze Li, Lin Feng, Jin He, Chengfeng Wang

**Affiliations:** 1 Pancreatic and Gastric Surgery Department, National Cancer Center/Cancer Hospital, Chinese Academy of Medical Sciences and Peking Union Medical College, 100021 Beijing, China; 2 State Key Laboratory of Molecular Oncology, National Cancer Center/Cancer Hospital, Chinese Academy of Medical Sciences and Peking Union Medical College, Beijing, 100021 China; 3 Department of Surgery, Sol Goldman Pancreatic Cancer Research Center, Johns Hopkins University School of Medicine, Baltimore, MD 21287 USA

**Keywords:** Pancreatic cancer, ANP32E, Proliferation, Migration, β-Catenin

## Abstract

**Background:**

Pancreatic cancer is a malignant tumor with high mortality. Acidic nuclear phosphoprotein 32 family member E (ANP32E), a specific H2A.Z chaperone, has been shown to contribute to breast cancer development. However, the significance of ANP32E in pancreatic cancer is poorly understood. This study aimed to investigate the role of ANP32E in pancreatic cancer.

**Methods:**

The expression of ANP32E in 179 pancreatic cancer tissues and 171 normal tissues, and the correlation between ANP32E expression and patients’ survival were analyzed from the TCGA database. ANP32E was over-expressed and silenced using lentivirus. siRNA was used to knock down β-catenin. CCK8, colony formation, cell cycle and transwell experiments were performed to determine cell proliferation and migration. qRT-PCR and Western blot were conducted to detect mRNA and protein expression.

**Results:**

ANP32E was up-regulated in pancreatic cancer tissues and cells. Up-regulation of ANP32E predicted poor prognosis in pancreatic cancer patients. Lentivirus-mediated knockdown of ANP32E suppressed the proliferation, colony growth and migration of PANC1 and MIA cells. By contrast, ANP32E over-expression promoted the proliferation and migration of both cells. In addition, ANP32E accelerated the cell cycle progression in PANC1 and MIA cells. Molecular experiments showed that ANP32E activated β-catenin/cyclin D1 signaling. Silencing of β-catenin reduced cell proliferation and migration in ANP32E over-expressed cells.

**Conclusion:**

Our results propose that ANP32E functions as an oncogene in pancreatic cancer via activating β-catenin.

**Supplementary Information:**

The online version contains supplementary material available at 10.1186/s12885-020-07556-z.

## Background

Pancreatic ductal adenocarcinoma (PDAC) is the commonest malignancy of the pancreas, with approximately 367,000 newly diagnosed cases and 359,000 PDAC-related deaths worldwide in 2015 [1]. The major risk factors for pancreatic cancer include tobacco smoking, alcohol use, type II diabetes, and chronic pancreatitis [2]. Various studies have shown that genetic variants of KRAS, CDKN2A, TP53 and SMAD4 contribute to the development of pancreatic cancer. However, based on current information from the pharmacy companies or clinical studies, none of these genes are easily targeted. Thus, identifying novel driver genes may help develop effective drugs against this deadly disease.

H2A.Z is a pivotal histone variant involved in regulating transcriptional activation or inhibition. It is essential for the survival and development of Drosophila and mouse [3, 4]. As a specific H2A.Z histone chaperon, acidic nuclear phosphoprotein 32 family member E (ANP32E) removes the H2A.Z from transcription region of the target genes to regulate their expression [5, 6]. ANP32E plays an important role in cerebellar development and synaptogenesis [7, 8]. Dys-regulation of ANP32E contributes to the migration and invasion of breast cancer cells [9]. Over-expression of ANP32E promotes triple-negative breast carcinogenesis through up-regulating E2F1 [10]. However, the involvement of ANP32E in pancreatic cancer is unclear.

Here in this study, we explored the role of ANP32E in pancreatic cancer. ANP32E was up-regulated in pancreatic cancer tissues. Up-regulation of ANP32E promoted the proliferation, colony growth and migration of pancreatic cancer cells. Cell cycle was regulated by ANP32E. Mechanistically, ANP32E induced the expression of β-catenin and its down-stream target cyclin D1. Silencing of β-catenin suppressed the proliferation and migration of ANP32E over-expressed PANC1 cells. Our study highlights the oncogenic role ANP32E in pancreatic cancer.

## Methods

### TCGA analysis of ANP32E expression in pancreatic patients

The transcript level of ANP32E in pancreatic cancer tissues and normal tissues, and the survival information of the patients were downloaded from The Cancer Genome Atlas (http://cancergenome.nih.gov) database. A total of 179 tumor tissues and 175 normal tissues were included to analyze the expression of ANP32E. A total of 89 patients with ANP32E high expression and 89 patients with low expression were included to determine the correlation between ANP32E expression and patients’ survival.

### Cell culture

Human normal ductal epithelial cells of the pancreas HPDE and pancreatic cancer cells AsPC1, PANC1 and MIA were obtained from ATCC. The cells were cultured in Dulbecco modified Eagle’s medium (DMEM, Hyclone), which contained 10% fetal bovine serum (FBS, Gibco) and 1% penicillin and streptomycin (Corning). Cell culture was maintained in a 37 °C incubator with 5% CO_2_.

### ANP32E knockdown

Lentivirus vector system pGCSIL-GFP (with a GFP marker), Helper2.0 (VSVG element) and pHelper1.0 (gag/pol element) were used to knock down ANP32E in PANC1 and MIA cells. Targeting sequence of the Ctrl, ANP32E#1 and ANP32E#2 was 5′-TTCTCCGAACGTGTCACGT-3′, 5′-GTCCACCGGAAGGATATGA-3′ and 5′-GCCTCTCATACTTAATGAA-3′. The lentivirus was packaged by co-transfecting the pGCSIL-GFP (20 μg), Helper2.0 (10 μg) and pHelper1.0 (15 μg) to 293FT cells. Knockdown efficiency was detected by qRT-PCR and Western blot.

### ANP32E over-expression

Lentivirus vector system pCDH, PSPAX2 and PDM2G was used to over-express ANP32E in PANC1 and MIA cells. Coding sequence of ANP32E (NM_030920) was cloned into the pCDH vector, which contained a GFP marker. Lentivirus was packaged by co-transfecting the pCDH (21 μg), PSPAX2 (16 μg) and PDM2G (10.5 μg) to 293FT cells. Over-expression efficiency was detected by qRT-PCR and Western blot.

### β-Catenin interference

The siRNAs against CTNNB or negative control were synthesized from GenePharma. The transfection of siRNAs (30 nM) were conducted using RNAimax (Invitrogen), following the the manufacturer’s instructions. The targeted sequence of siRNA was as followed: siCTNNB, 5′-CCCACTAATGTCCAGCGTT-3′; and siCtrl, 5′-TTCTCCGAACGTGTCACGT-3′.

### RNA extraction and quantitative real-time polymerase chain reaction (qRT-PCR)

PANC1 and MIA cells were lysed using Trizol regent. Total RNA was extracted from the cells and 1 μg of the RNA was subjected to reversed transcription reaction with M-MLV reverse transcriptase (Promega). Quantification of the cDNA (1/50 of the reversed product) was performed in triplicate with SYBR master mixture on the biorad machine. The qPCR primer sequence was as followed: ANP32E forward, 5′-TGCCTGTGTGTCAATGGGG-3′, and reverse, 5′-GCAGAGCTTCTACTGTACTGAGA-3′; and GAPDH forward, 5′-TGACTTCAACAGCGACACCCA-3′, and reverse, 5′-CACCCTGTTGCTGTAGCCAAA-3′. The expression of ANP32E was normalized to GAPDH. Transcript of ANP32E and GAPDH was 250 bp and 225 bp, respectively.

### Western blot

Total protein was extracted from PANC1 and MIA cells with RIPA lysis buffer (Beyotime). BCA experiment (Beyotime) was conducted to measure the protein concentration. 50 μg of the protein was separated on 10–12% SDS-PAGE gels. Subsequently, the proteins were transferred onto PVDF membranes. 5% skim milk was used to block the membranes for 1 h at room temperature. The membranes were incubated with indicated primary antibodies at 4 °C overnight. Antibody against ANP32E (ab5993, 1:1000 dilution) was from Abcam. β-actin primary antibody (sc47778, 1:3000 dilution) and the secondary antibodies (sc2004 and sc2005, 1:5000 dilution) were obtained from SantaCruz.

### CCK8 assay

Cell proliferation was determined by CCK8 assay. Briefly, ANP32E silenced or over-expressed and the control cells were seeded in triplicate into 96-well plates at the density of 2000 cells per well. The cells were cultured for 4 days. 1, 2, 3 and 4 days later, 10% the CCK8 regent (YEASEN) was added into each well and the plates were maintained at 37 °C. 3 h later, the OD value at 450 nm was measured on the micro-plate machine. The cell viability was normalized to the OD value of the first day.

### Colony formation assay

A total of 500 shCtrl, shANP32E#1 and shANP32E#2, OE-Ctrl and OE-ANP32E PANC1 and MIA cells were seeded in triplicate into the 6-well plates. 10 days later, colonies were formed and the cell culture was removed. After washed by PBS for three times, the colonies were fixed by methanol and stained by crystal violet. Subsequently, the plates were washed by clean water and dried at room temperature. Then the colonies were photographed by the camera.

### Cell cycle analysis

PI staining was used to analyze the cell cycle in PANC1 and MIA cells. Indicated cells were seeded in triplicate in 6-well plates. 2 days later, the cells were re-suspended in FBS free culture medium and fixed by 70% ethanol. After stained by PI regent, flow cytometer was used to analyze the cell cycle distribution in PANC1 and MIA cells.

### Transwell assay

A total of 3 × 10^4^ PANC1 or MIA cells in 200ul FBS free culture medium were seeded in triplicate onto the upper surface of 8.0-μm filter migration chambers. The lower compartment contained 500ul DMEM medium with 10% FBS. 24 h later, the cells attached on the upper surface were removed by cotton tips. The cells on the lower surface were fixed by methanol and stained by crystal violet. The images in three fields were photographed under a microscope (OLYMPUS, 200x).

### Statistical analysis

All the experiments were conducted for three independent repeats. GraphPad prism software was used to analyze the data as shown in the Figure. Students’t test was used to determine the difference between two groups. One-way ANOVA was used to determine the difference among more than two groups. Statistical difference was considered significant when *p* < 0.05.

## Results

### ANP32E is over-expressed in pancreatic cancer tissues and predicts the survival of patients

**Fig. 1:**
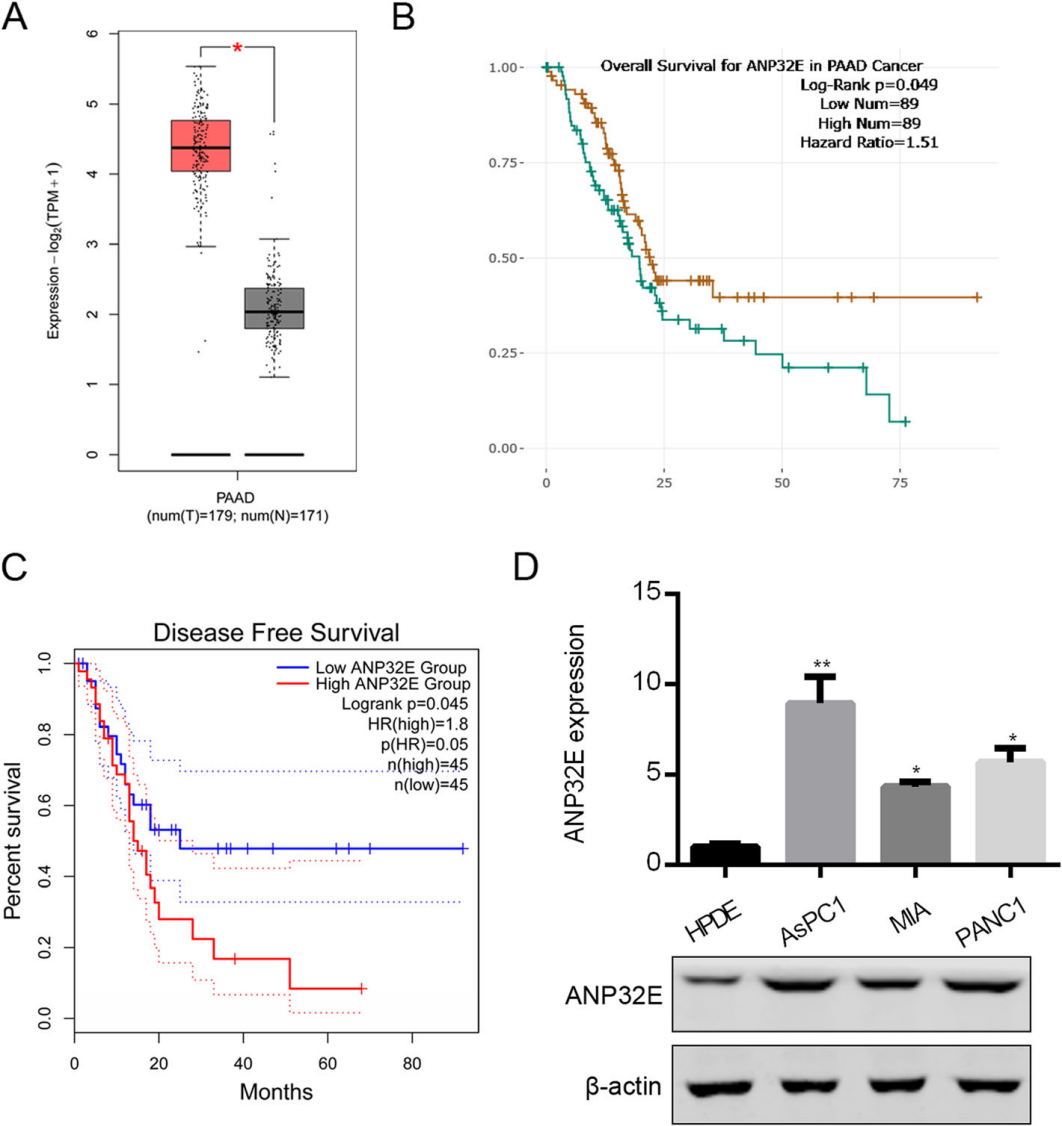
Clinical relevance of ANP32E in pancreatic cancer patients. **a** The transcript abundance of ANP32E in PAAD (*n* = 179, red box) and normal tissues (*n* = 175, gray box) was analyzed from TCGA database. *p* < 0.001. **b** PAAD patients were divided into ANP32E high expression (*n* = 89, green line) and low expression (n = 89, yellow line) group. Overall survival for ANP32E was analyzed. *p* = 0.049. **c** PAAD patients were divided into ANP32E high expression (*n* = 45, red line) and low expression (n = 45, blue line) group. Disease free survival for ANP32E was analyzed. *p* = 0.045. **d** qRT-PCR and Western blot analysis of ANP32E in human normal ductal epithelial cells of the pancreas HPDE and pancreatic cancer cells AsPC1, PANC1 and MIA. *p < 0.05. **p < 0.01. Full-length gels are presented in Supplementary Figure 2

To study the clinical relevance of ANP32E in pancreatic cancer, we downloaded the transcript abundance of ANP32E and pancreatic adenocarcinoma (PAAD) patients’ survival information from TCGA database. We found that ANP32E was over-expressed in PAAD tissues comparing to normal pancreatic tissues (Fig. 1a). PAAD patients were divided into two groups: ANP32E low expression and high expression group. Survival curve showed that ANP32E high expression patients exhibited poorer overall and disease free survival than those with ANP32E low expression (Fig. 1b and c). We also checked the abundance of ANP32E in human normal ductal epithelial cells of the pancreas HPDE and pancreatic cancer cells AsPC1, PANC1 and MIA. qRT-PCR and Western blot results showed HPDE cells had lowest ANP32E expression comparing with AsPC1, PANC1 and MIA cells (Fig. 1d). These results suggest that ANP32E is correlated with the disease progression of PAAD.

### ANP32E promotes the proliferation and growth of pancreatic cancer cells

To determine whether ANP32E functions as an oncogene in pancreatic cancer, we used lentivirus to knock down ANP32E in pancreatic cancer cells PANC1 and MIA. qRT-PCR and Western blot results showed that ANP32E was efficiently knocked down in PANC1 and MIA cells (Supplementary Fig. 1A and 1B). We also used lentivirus vector system to over-express ANP32E. Comparing with Ctrl cells, ANP32E over-expressed cells had significantly higher mRNA and protein abundance of ANP32E (Supplementary Fig. 1C and 1D). These results suggest that ANP32E is efficiently silenced and over-expressed in PANC1 and MIA cells.

**Fig. 2:**
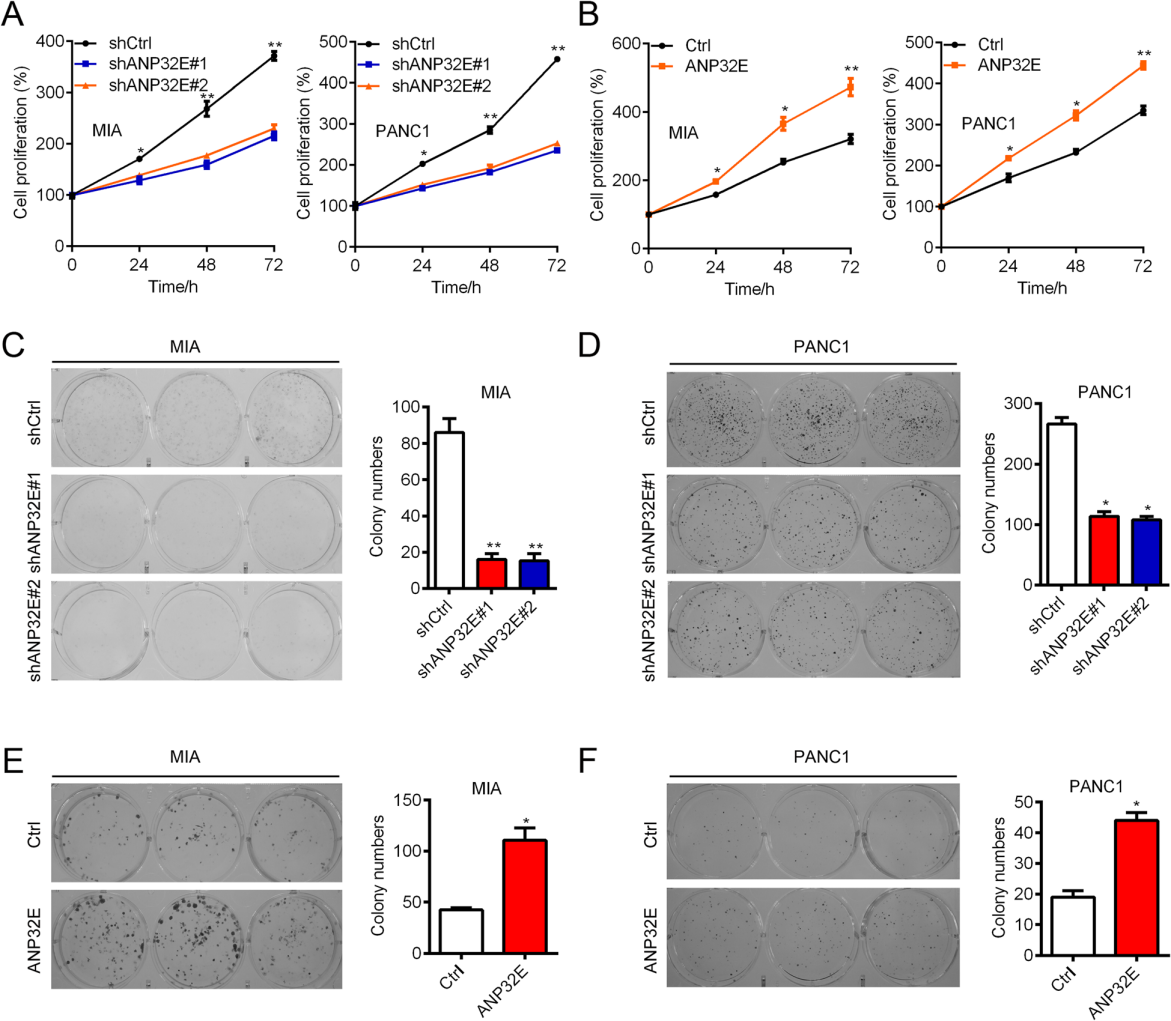
ANP32E promotes the proliferation and colony growth of pancreatic cancer cells. **a** shCtrl, shANP32E#1 and shANP32E#2 MIA and PANC1 cells were subjected to CCK8 analysis of cell proliferation. *p < 0.05. **p < 0.01. **b** Ctrl and ANP32E over-expressed PANC1 and MIA cells were subjected to CCK8 analysis of cell proliferation. *p < 0.05. **p < 0.01. **c** and **d** shCtrl, shANP32E#1 and shANP32E#2 MIA (**c**) and PANC1(D) cells were subjected to colony formation assays. Left, colony images. Right, quantification result. *p < 0.05. **p < 0.01. **e** and **f** Ctrl and ANP32E over-expressed MIA (**e**) and PANC1 (**f**) cells were subjected to colony formation assays. Left, colony images. Right, quantification result. *p < 0.05. Results are mean ± SEM (*n* = 3)

To examine the effect of ANP32E on cell proliferation, CCK8 assays were performed in ANP32E knockdown and over-expressed cells. We found that down-regulation of ANP32E suppressed the proliferation of PANC1 and MIA cells (Fig. 2a). By contrast, up-regulation of ANP32E enhanced the proliferation of both cells (Fig. 2b). Consistently, ANP32E silencing and over-expression reduced and enhanced the colony formation capacity of PANC1 and MIA cells, respectively (Fig. 2c-f). Collectively, ANP32E functions as an oncogene in pancreatic cancer.

### ANP32E promotes the migration of pancreatic cancer cells

**Fig. 3:**
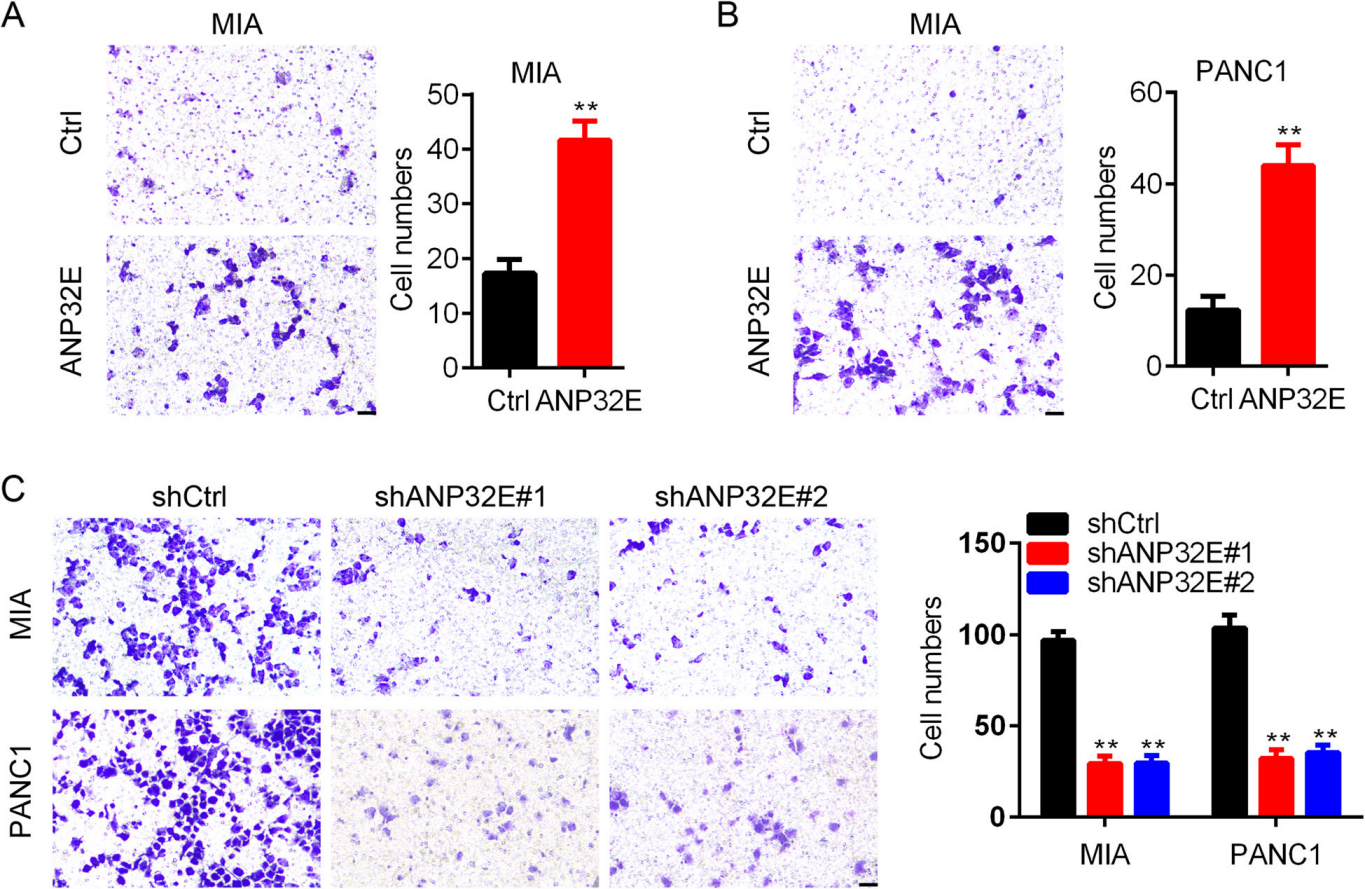
ANP32E promotes the migration of pancreatic cancer cells. **a** Transwell assay was performed to analyze the migration in Ctrl and ANP32E over-expressed MIA cells. Left, cell images. Right, quantification result. **p < 0.01. **b** Transwell assay was performed to analyze the migration in Ctrl and ANP32E over-expressed PANC1 cells. Left, cell images. Right, quantification result. **p < 0.01. **c** Transwell assay was performed to analyze the migration in shCtrl, shANP32E#1 and shANP32E#2 MIA and PANC1 cells. Left, cell images. Right, quantification result. **p < 0.01. Results are mean ± SEM (*n* = 3). Scale bar, 200 um

Next, we assessed the role of ANP32E on pancreatic cancer cell migration. Transwell assay was conducted to evaluate the migration capacity in ANP32E knockdown and over-expressed PANC1 and MIA cells. The results showed that ANP32E over-expression promoted the migration in PANC1 and MIA cells (Fig. 3a and b). Moreover, comparing to shCtrl PANC1 and MIA cells, shANP32E#1 and shANP32E#2 cells exhibited reduced migration capacity (Fig. 3c). Collectively, ANP32E enhanced the migration of pancreatic cancer cells.

### ANP32E promotes the cell cycle progression in pancreatic cancer cells

**Fig. 4:**
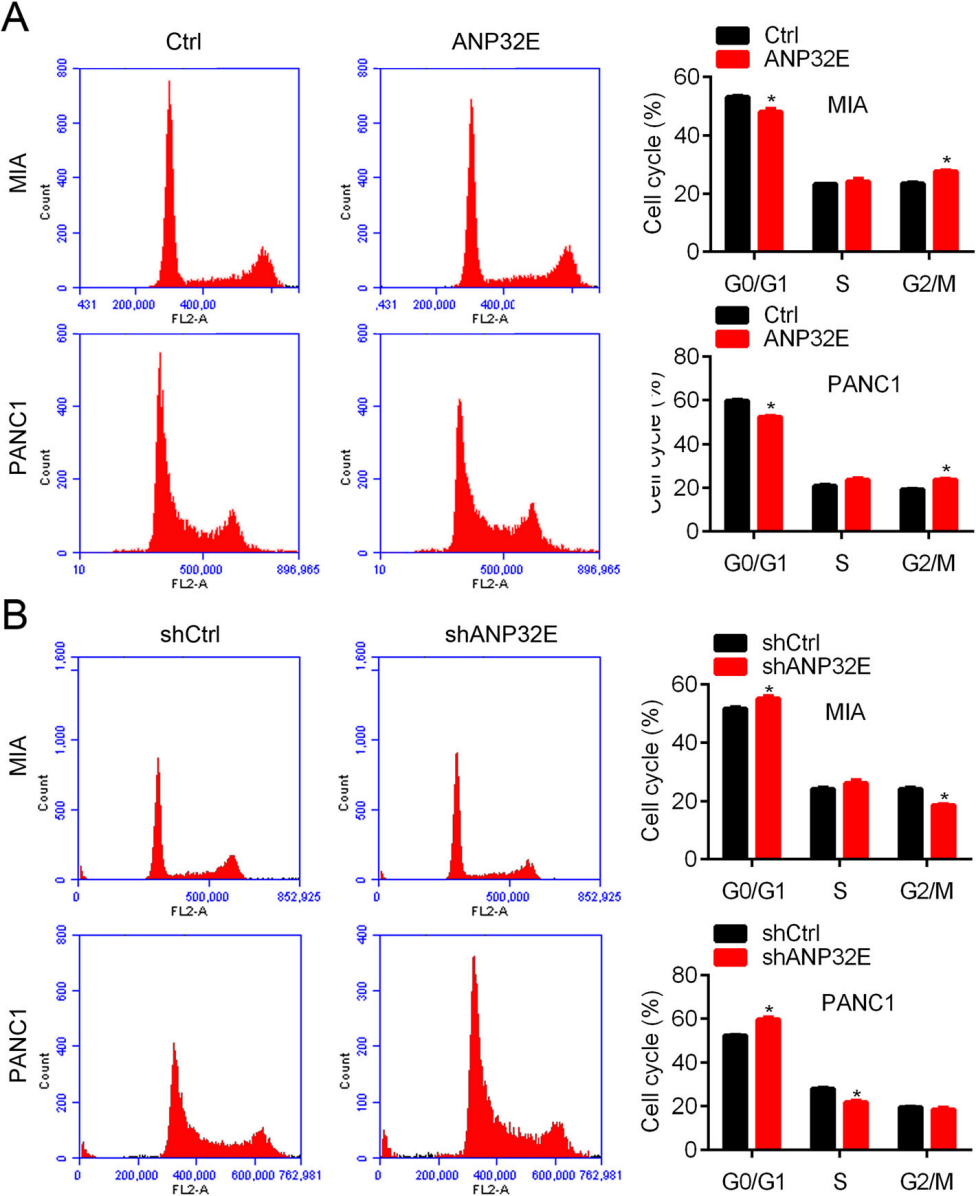
ANP32E regulates cell cycle in pancreatic cancer cells. **a** Cell cycle distribution was detected by PI staining in Ctrl and ANP32E over-expressed MIA and PANC1 cells. Left, cell cycle images. Right, quantification result. *p < 0.05. **b** Cell cycle distribution was detected by PI staining in shCtrl and shANP32E MIA and PANC1 cells. Left, cell cycle images. Right, quantification result. *p < 0.05. Results are mean ± SEM (n = 3)

Accelerated cell cycle process is a hallmark of cancer cell. We examined the effect of ANP32E on cell cycle distribution. PANC1 and MIA cells infected with ANP32E over-expression lentivirus were stained with PI and subjected to flow cytometer analysis of cell cycle. Ectopic expression of ANP32E led to reduced G0 phase and increased S and G2/M phase (Fig. 4a). By contrast, ANP32E knockdown resulted in increased G0 phase and decreased S and G2/M phase (Fig. 4b). These results indicate that ANP32E may promote cell cycle progression.

### ANP32E up-regulates β-catenin to promote the proliferation and migration of pancreatic cancer cells

**Fig. 5:**
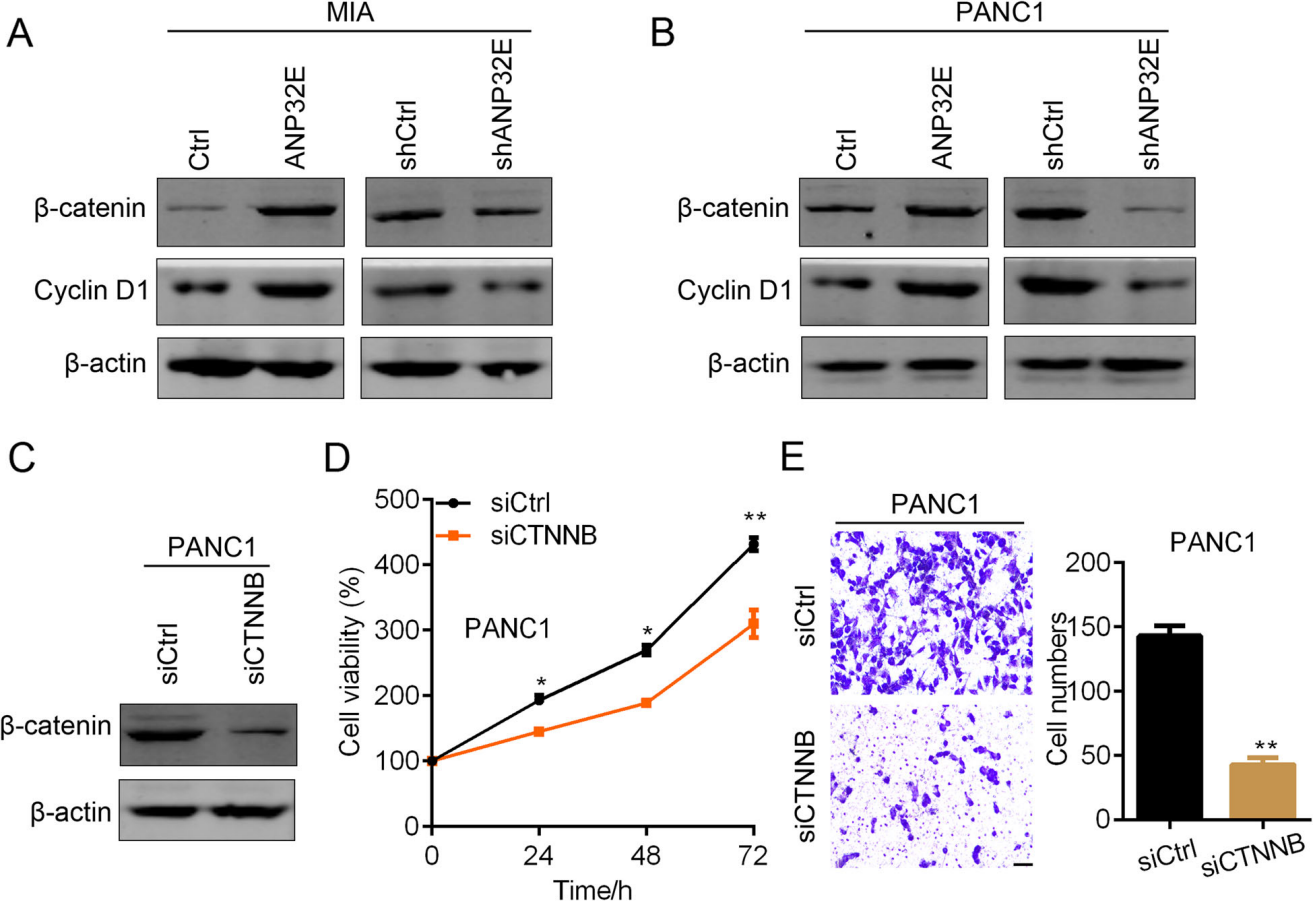
ANP32E activates β-catenin to promote the proliferation and migration of pancreatic cancer cells. **a** and **b** Western blot results of β-catenin and cyclin D1 in Ctrl and ANP32E over-expressed, shCtrl and shANP32E MIA (**a**) and PANC1 (**b**) cells. **c** Western blot results of β-catenin in siCtrl and siCTNNB PANC1 cells with highly expressed ANP32E. **d** siCtrl and siCTNNB PANC1 cells were subjected to CCK8 analysis of cell proliferation. *p < 0.05. **p < 0.01. **e** siCtrl and siCTNNB PANC1 cells were subjected to Transwell analysis of migration. **p < 0.01. Results are mean ± SEM (n = 3). Full-length gels are presented in Supplementary Figure 2. Scale bar, 200 um

We attempted to explore the down-stream targets of ANP32E using Western blot assays. We found the expression of β-catenin and cyclin D1 was increased in ANP32E over-expressed MIA cells (Fig. 5a). Oppositely, ANP32E down-regulation inhibited the expression of β-catenin and cyclin D1 (Fig. 5a). Consistent results were observed in ANP32E over-expressed and silenced PANC1 cells (Fig. 5b). To evaluate whether β-catenin contributes to the oncogenic role of ANP32E in pancreatic cancer, we knocked down CTNNB, which encodes β-catenin, in ANP32E over-expressed PANC1 cells. Western blot showed that β-catenin was efficiently silenced in PANC1 cells (Fig. 5c). Down-regulation of β-catenin significantly repressed the proliferation and migration of PANC1 cells (Fig. 5d and e). These results suggest that ANP32E promotes pancreatic cancer through potentiating β-catenin signaling.

## Discussion

Pancreatic cancer is a lethal malignancy. Once diagnosed, the disease progresses fast and 5-year survival rate is very low. Identifying novel oncogenes or tumor suppressors may help develop effective drugs to cure this malignancy. In this study, we found that ANP32E was an oncogene in pancreatic cancer. High expression of ANP32E was found in PAAD tissues and predicted poorer survival of PAAD patients. In vitro, ANP32E promoted the proliferation, colony formation and migration of pancreatic cancer cells. ANP32E also regulated cell cycle in these cells. β-catenin/cyclin D1 signaling was positively regulated by ANP32E. Inhibition of β-catenin reduced the proliferation and migration of pancreatic cancer cells. These findings indicate that ANP32E promotes pancreatic cancer through regulating β-catenin/cyclin D1 signaling.

Acidic nuclear phosphoprotein 32 family member E (ANP32E) is a specific H2A.Z histone chaperon. It regulates the expression of target genes by removing H2A.Z from transcriptional region of the genes [5, 6]. ANP32E also plays a pivotal role in nucleosome reorganization and DNA repair by removing H2A.Z from DNA double-strand breaks [11]. Several studies have investigated the role of ANP32E in cancer development. For example, Li et.al showed that miR-141 repressed the proliferation and migration of breast cancer cells and the expression of ANP32E. Knockdown of ANP32E reduced cell growth [9]. Thus, ANP32E contributes to the tumor suppressive role of miR-141 in breast cancer. In addition, ANP32E promotes the development of triple-negative breast cancer via transcriptionally activating E2F1 [10]. These results indicate that ANP32E may be an oncogene in cancers. In this study, we observed that ANP32E was up-regulated in PAAD tissues. ANP32E expression was also inversely correlated with the prognosis of PAAD patients. In vitro, ANP32E over-expression promoted the proliferation, colony formation and migration of PANC1 and MIA cells. Opposite results were found in ANP32E silenced cells. Furthermore, ANP32E promoted cell cycle progression in PANC1 and MIA cells. Our results propose that ANP32E functions as an oncogene in pancreatic cancer.

Due to alterations of any component of Wnt/β-catenin signaling pathway, it is frequently activated in various cancers, including colorectal cancer, hepatocellular carcinoma and pancreatic cancer [12–14]. In brief, Wnt ligand binds to Frizzled receptor family, leading to inactivation of adenomatous polyposis coli (APC) or axin, which in turn promotes the cytoplasmic accumulation and nuclear localization of β-catenin. β-catenin can also be regulated by other factors to promote cancer development. For instance, TYRO3 promotes the proliferation and metastasis of gastric cancer cells through activation of Wnt/β-catenin signaling pathway [15]. LncCCAT1 enhances the function of breast cancer stem cell via activating WNT/β-catenin signaling [16]. β-catenin is a transcriptional factor that interacts with TCF/LEF factors to activate the expression of target genes. The well-known downstream targets for β-catenin are c-myc and cyclin D1 [17, 18]. However, the correlation between ANP32E and β-catenin is unclear in pancreatic cancer. Here, our molecular experiments showed that ANP32E up-regulated β-catenin and its downstream target cyclin D1. We also checked other oncogenes, such as pyruvate kinase M2 (PKM2) and hexokinase 2 (HK2), or tumor suppressors, including P53 and PTEN. No difference was observed after knocking down ANP32E. We also analyzed the role of β-catenin in ANP32E over-expressed cells. The results showed that knockdown of β-catenin suppressed the growth and migration of pancreatic cancer cells. Taken together, ANP32E promotes pancreatic cancer through activating β-catenin.

## Conclusions

We propose that ANP32E is an oncogene in pancreatic cancer. ANP32E promotes the proliferation and migration of pancreatic cancer cells through up-regulation and activation of β-catenin/cyclin D1 signaling. Our findings suggest that ANP32E is a promising therapeutic target for PAAD.

## Supplementary Information

**Additional file 1.**

## Data Availability

All data generated or analyzed during this study are included in this published article.

## Abbreviations

ANP32E: Acidic nuclear phosphoprotein 32 family member E
PDAC: Pancreatic ductal adenocarcinoma
TCGA: The Cancer Genome Atlas
DMEM: Dulbecco modified Eagle’s medium
FBS: fetal bovine serum
qRT-PCR: Quantitative real-time polymerase chain reaction
APC: Adenomatous polyposis coli
