# Supplementary material for: Over-expression of ANP32E is associated with poor prognosis of pancreatic cancer and promotes cell proliferation and migration through regulating β-catenin
Source: BMC Cancer. 2020 Nov 4;20:1065. doi: 10.1186/s12885-020-07556-z (PMC7640479; doi:10.1186/s12885-020-07556-z)

**Fig. 1 ANP32E is efficiently knocked down and over-expressed in MIA and PANC1 cells.**


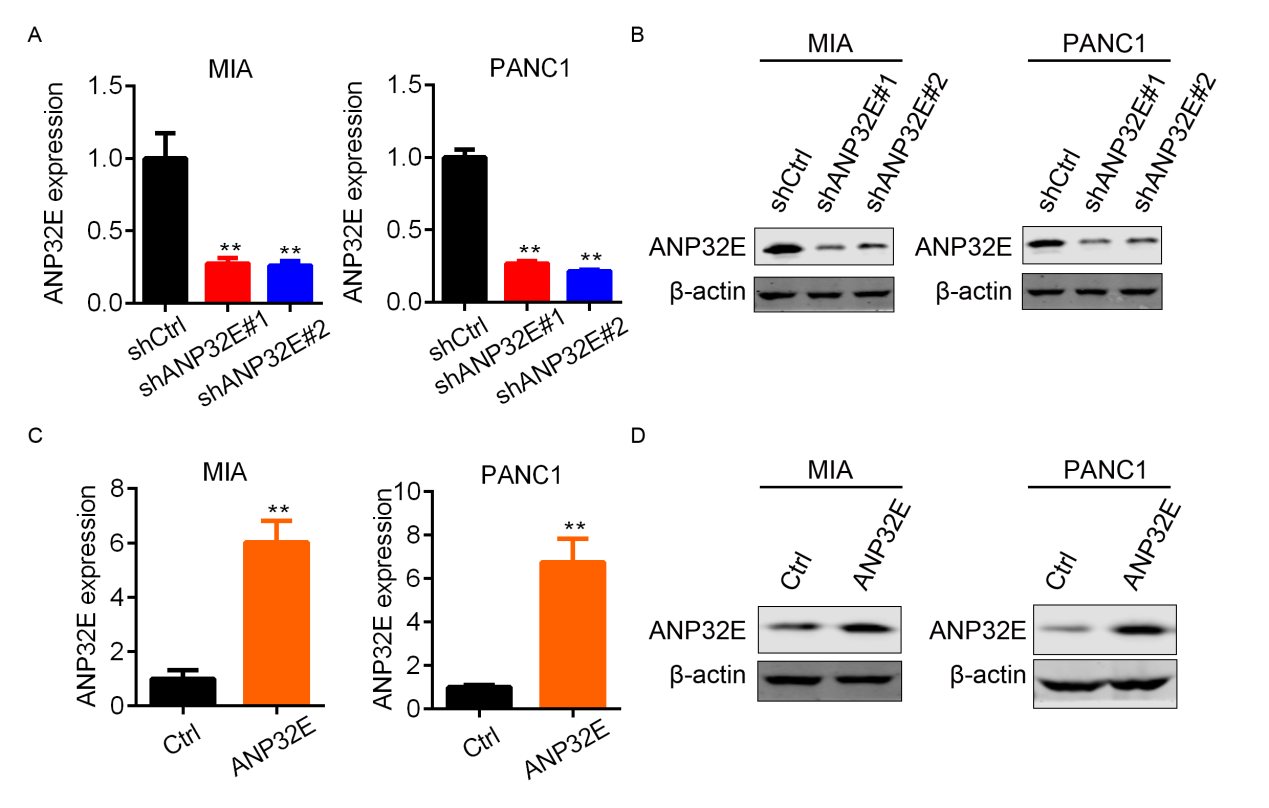


(A and B) qRT-PCR (A) and Western blot (B) analysis of ANP32E in shCtrl, shANP32E#1 and shANP32E#2 MIA and PANC1 cells. **p<0.01. (C and D) qRT-PCR (C) and Western blot (D) results of ANP32E in Ctrl and ANP32E over-expressed MIA and PANC1 cells. **p<0.01. Full-length gels are presented in Supplementary Figure 2.

**Fig. 2 Full-length gels.**


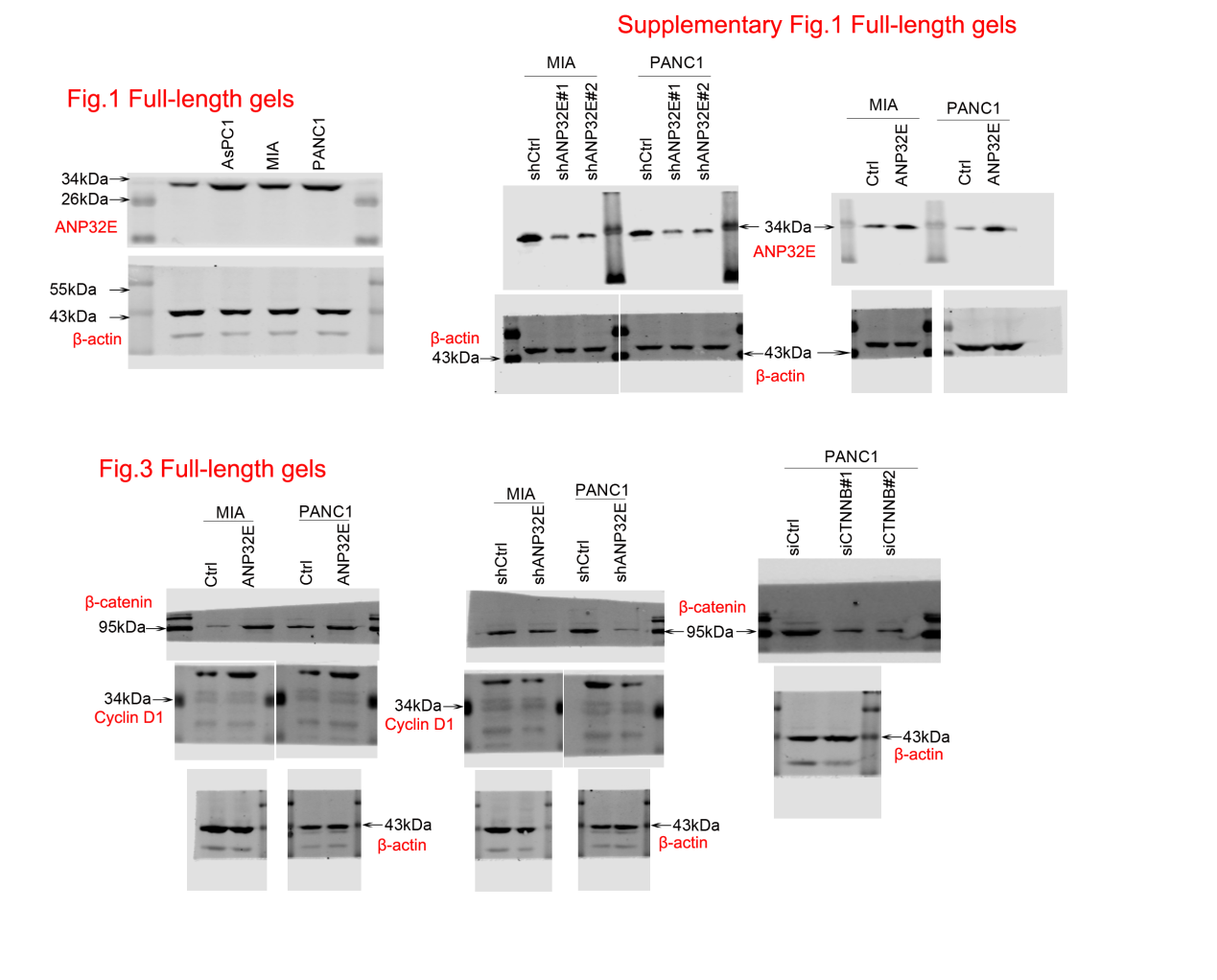

Supplement: Supplementary file 1 — Additional file 1. [file 12885_2020_7556_MOESM1_ESM.docx]
